# Jumihaidokuto (Shi-Wei-Ba-Du-Tang), a Kampo Formula, Decreases the Disease Activity of Palmoplantar Pustulosis

**DOI:** 10.1155/2016/4060673

**Published:** 2016-04-10

**Authors:** Megumi Mizawa, Teruhiko Makino, Chieko Inami, Tadamichi Shimizu

**Affiliations:** Department of Dermatology, Graduate School of Medicine and Pharmaceutical Sciences, University of Toyama, 2630 Sugitani, Toyama 930-0194, Japan

## Abstract

Palmoplantar pustulosis (PPP) is a chronic skin disease characterized by sterile intraepidermal pustules associated with erythematous scaling on the palms and soles. Jumihaidokuto is a traditional herbal medicine composed of ten medical plants and has been given to patients with suppurative skin disease in Japan. This study investigated the effect of jumihaidokuto on the disease activity in PPP patients (*n* = 10). PPP patients were given jumihaidokuto (EKT-6; 6.0 g per day) for 4 to 8 weeks in addition to their prescribed medications. The results showed that the palmoplantar pustular psoriasis area and severity index (PPPASI) was decreased after the administration of jumihaidokuto (*p* < 0.05). Therefore, Jumihaidokuto is seemingly effective against PPP.

## 1. Introduction

Palmoplantar pustulosis (PPP), also referred to as pustulosis palmaris et plantaris, is a chronic skin disease characterized by sterile intraepidermal pustules associated with erythematous scaling on the palms and soles [1]. Although PPP is a relatively common skin disease in Japan, the precise pathogenesis of PPP remains unknown. The standard therapy for PPP patients includes topical corticosteroids, topical vitamin D3 analogs, oral cyclosporine A, psoralen plus ultraviolet A therapy (PUVA), and narrowband ultraviolet- (UV-) B. However, clinicians often experience PPP that is refractory to these treatments. Kampo medicines, such as orengedokuto (Huang-Lian-Jie-Du-Tang), unseiin (Wen-Qing-Yin), and keishibukuryogan (Gui-Zhi-Fu-Ling-Wan) have been reported to be effective for PPP [2]. Jumihaidokuto (JHT, Shi-Wei-Ba-Du-Tang in Chinese) is a traditional herbal medicine that is composed of ten medicinal plants (Kikyo (*Platycodi Radix*), Saiko (*Bupleuri Radix*), Senkyu (*Chuanxiong Rhizoma*), Bukuryo (*Sclerotium Poriae Cocos*), Dokkatsu (*Angelicae pubescentis Radix*), Bofu (*Saposhnikoviae Radix*), Kanzo (*Glycyrrhizae Radix*), Keigai (*Schizonepetae Herba*), Shokyo (*Zingiberis Rhizoma*), and Bokusoku (*Quercus cortex*)) [3]. According to Kampo medicinal sources, Bokusoku (*Quercus cortex*) can be used in place of Ouhi (*Pruni Cortex*). JHT is often given to patients with certain skin diseases, such as eczema, trichophytia, and acne vulgaris [4]. Higaki et al. previously reported that JHT can effectively suppress acne rashes [5, 6]. In the present study, we evaluated the clinical effect of JHT for patients with PPP.

## 2. Materials and Methods

### 2.1. Subjects

The study included ten PPP patients (4 males and 6 females; age, 59–77 years; mean age, 66.0 years). A diagnosis of PPP was performed according to the clinical findings by experienced dermatologists. The patients had no other concomitant diseases.

### 2.2. Study Design

The study was performed as a prospective self-controlled trial. These PPP patients were given JHT (EKT-6; 6.0 g per day; Kracie Holdings, Ltd., Tokyo, Japan) before meals, two times a day, for 4 to 8 weeks in addition to their prescribed medications, such as topical corticosteroids and oral antihistamines (Table 1). The patients were not allowed to take any other medication during the study period. Clinical assessments were performed at week 0 and week 4 or 8. This study was approved by the Human Subjects Committee, University of Toyama (approval number 25-95). All patients provided their written informed consent in accordance with the ethical guidelines set forth in the 1975 Declaration of Helsinki.

### 2.3. Assessment of the Disease Activity

The disease severity of PPP was evaluated using the palmoplantar pustular psoriasis area and severity index (PPPASI). The PPPASI score was calculated as described below. Erythema (E), pustules (I), and desquamation (D) were evaluated on a scale of 0 to 4, while the area was evaluated on a scale of 0 to 6. The following formula was used: PPPASI score = (E + I + D) × area × 0.2 (right palm) + (E + I + D) × area × 0.2 (left palm) + (E + I + D) × area × 0.3 (right sole) + (E + I + D) × area × 0.3 (left sole). The PPPASI score can vary from 0 (absence of disease) to 72 (most severe palmoplantar psoriasis possible) [7]. In addition, the percent change of the PPPASI score for each patient before and after JHT treatment was also assessed.

### 2.4. Statistical Analysis

Data are presented as the mean values plus the standard error of the mean ± S.D. All statistical analyses were performed using paired *t*-test. A *p* value of less than 0.05 was considered to be statistically significant.

## 3. Results

**Figure 1:**
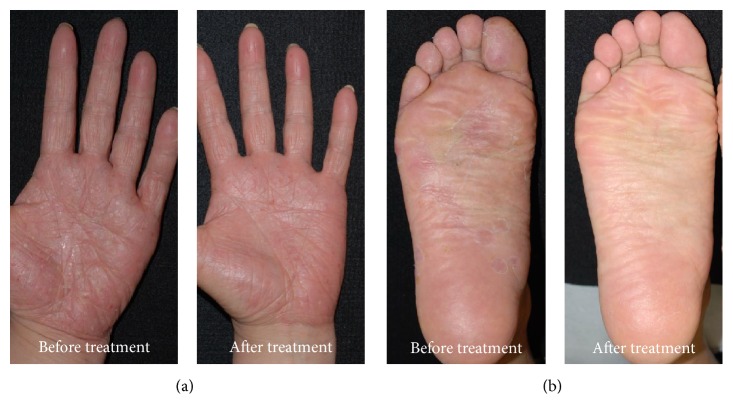
(a) Case 1. Multiple pustules over the left palm with scaling and erythema were markedly improved after 6-week treatment. (b) Case 7. Hyperkeratotic lesions over the right sole disappeared after 4-week treatment.

**Figure 2:**
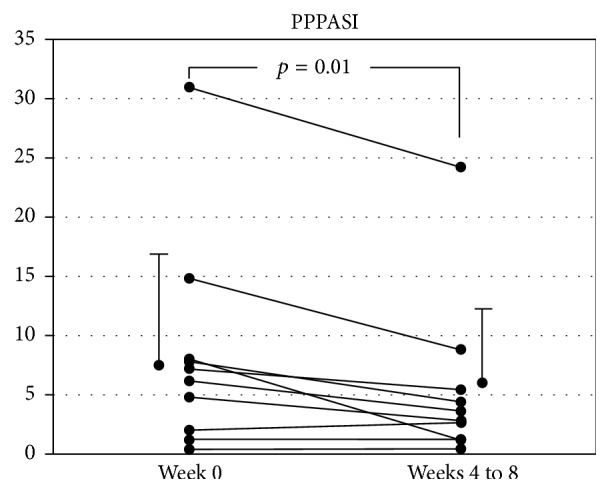
The PPPASI in patients with PPP before and after treatment with JHT. These indices were significantly decreased after JHT treatment (*p* = 0.01).

Ten PPP patients were given JHT for 4 to 8 weeks. Seven out of 10 PPP patients showed an improvement in their clinical findings (Table 1). In most of these patients, the number of pustules on the palms and soles markedly decreases (Figure 1(a)). In addition, some patients showed a disappearance of hyperkeratotic lesions (Figure 1(b)). The average PPPASI of all patients was 8.34 ± 9.00 before JHT treatment. Four or 8 weeks after the administration of JHT, the average PPPASI significantly decreased (5.46 ± 7.02, *p* < 0.01; Figure 2). No adverse event was observed during the study period.

## 4. Discussion

Traditional herbal medicine, also known as Kampo medicine in Japan, has a long history and plays a role in the prevention and treatment of various inflammatory skin diseases. Kampo medicines have been occasionally used as a treatment for PPP and certain Kampo herbal drugs are known to be effective for PPP, even when the symptom is resistant to standard treatment. For instance, orengedokuto (Huang-Lian-Jie-Du-Tang) is effective for erythematous lesions of PPP [2]. Unseiin (Wen-Qing-Yin) and keishibukuryogan (Gui-Zhi-Fu-Ling-Wan) can improve hyperkeratotic lesions of PPP [2]. Furthermore, we recently reported that the administration of JHT markedly decreased the number of pustules on the palms and soles of a PPP patient [8]. The present study demonstrated that JHT can significantly decrease the disease activity of PPP according to the PPPASI. Although there has been no reported case regarding Kampo treatment for PPP in the English literature, we found 2 clinical studies in the Japanese literature which described the effect of Kampo medicines for PPP patients. One study described that the treatment of JHT was effective for 64.9% of PPP patients [9]. Another study reported that the administration of JHT for 12 weeks resulted in a mild improvement in 49.9% of PPP patients [10]. These reports are considered to be compatible with the result of the present study. In contrast, in the present study, three patients showed no improvement in their PPPASI score. This may be because the PPPASI scores before JHT treatment in these 3 patients were very low compared to those in the other patients. In addition, JHT is known to have side effects, including pseudoaldosteronism and myopathy, although no adverse events were observed in all patients during the present study.

JHT is composed of ten medicinal plants. Among them,* Bupleuri Radix* and* Glycyrrhizae Radix* have antisuppurative and anti-inflammatory activities. Furthermore,* Platycodi Radix* is known to drain pus [11]. The JHT formula used in this study includes* Pruni Cortex,* but not* Quercus Cortex*.* Quercus Cortex* is considered to be effective for the “*Okestu*” symptom, which appears to correspond to blood stasis [12]. In contrast,* Pruni Cortex* has antisuppuration and anti-inflammatory activities and a drainage effect of pus similar to* Bupleuri Radix* and* Glycyrrhizae Radix*. In addition a recent study demonstrated that an extract of* Pruni Cortex* possessed an estrogen-like effect. Estrogen can act as an antagonist of androgen; therefore,* Pruni Cortex* may reduce sebum secretion via the similar function of estrogen. This hypothesis may explain the function of JHT for acne vulgaris [3]. Taken together, the medical actions of* Bupleuri Radix*,* Glycyrrhizae Radix,* and* Pruni Cortex*, including antisuppuration, anti-inflammation, and drainage of pus, may induce an improvement in the symptom of PPP.

## 5. Conclusions

The present study demonstrated that JHT can decrease the disease activity of PPP, and JHT is considered to be a useful treatment option in patients with PPP.

## Figures and Tables

**Table 1:** Characteristics and evaluation of clinical symptoms in patients treated with JHT.

Case	Age (y)	Sex	Prescribed medications	Period (weeks)	PPPASI (before treatment)	PPPASI (after treatment)	PPPASI improvement (%)
1	59	Female	Topical corticosteroid, oral antihistamine, oral vitamin H	6	31	24.2	21.9

2	61	Female	Topical corticosteroid, topical vitamin D3	6	6.2	3.6	41.9

3	66	Male	Topical corticosteroid, topical vitamin D3	8	7.2	5.4	25

4	73	Female	Topical corticosteroid, oral antihistamine, oral vitamin H	4	4.8	2.8	41.7

5	66	Female	Topical corticosteroid, topical vitamin D3	8	7.8	4.4	43.6

6	63	Male	Topical corticosteroid, oral antihistamine, oral vitamin H	8	14.8	8.8	40.5

7	63	Male	Topical corticosteroid, oral antihistamine	4	8	1.2	85

8	77	Male	Topical corticosteroid, oral vitamin H	8	1.2	1.2	0

9	70	Female	Topical corticosteroid, oral vitamin H	5	0.4	0.4	0

10	59	Female	Topical corticosteroid, oral antihistamine, oral vitamin H	4	2	2.6	−30

## Abbreviations

PPP: Palmoplantar pustulosis
JHT: Jumihaidokuto.
